# Extrapolating Dynamic Leidenfrost Principles to Metallic Nanodroplets on Asymmetrically Textured Surfaces

**DOI:** 10.1038/srep11769

**Published:** 2015-06-30

**Authors:** Joseph E. Horne, Nickolay V. Lavrik, Humberto Terrones, Miguel Fuentes-Cabrera

**Affiliations:** 1 Department of Physics, Applied Physics & Astronomy, Rensselaer Polytechnic Institute, 110 Eighth Street, Troy, 12180 NY, USA; 2 Center for Nanophase Materials Sciences, Oak Ridge National Laboratory, Oak Ridge, TN, 37831, USA; 3 Computer Science and Mathematics Division, Oak Ridge National Laboratory, Oak Ridge, TN, 37831, USA

## Abstract

In an effort to enhance our knowledge on how to control the movement of metallic nanodroplets, here we have used classical molecular dynamics simulations to investigate whether Cu nanostructures deposited on nanopillared substrates can be made to jump at desired angles. We find that such control is possible, especially for Cu nanostructures that are symmetric; for asymmetric nanostructures, however, control is more uncertain. The work presented here borrows ideas from two seemingly different fields, metallic droplets and water droplets in the dynamic Leidenfrost regime. Despite the differences in the respective systems, we find common ground in their behavior on nanostructured surfaces. Due to this, we suggest that the ongoing research in Leidenfrost droplets is a fertile area for scientists working on metallic nanodroplets.

Controlling the movement of nanoscale objects can have significant applications in a variety of fields^1^. For example, a swift electron beam^2^ and a dynamic magnetic field generator^3^ have been recently used to control the movement of Au and iron oxide nanoparticles, respectively. The former technique suggests ways to create nanometer-size traps, whereas the latter demonstrates a novel methodology to control apoptosis. Photothermal activation of Al nanoparticles with a xenon lamp has also been used to control the motion of ejected Al nanodroplets, which could lead to innovations in combustion and propulsion^4^. A few years ago, employing theoretical techniques, we proposed that laser-induced dewetting of metallic nanostructures could be used to control the magnitude of the velocity of ejected metallic nanodroplets^5^. Our work was inspired by a previous experimental work by Habenicht *et al*.^6^.

In 2005, Habenicht *et al.*^6^, showed that Au droplets could be ejected when triangular Au nanostructures deposited on silica or graphite were heated with nanosecond laser pulses. The ejection is due to the near instantaneous melting of the nanostructure which, to minimize its surface energy, rapidly contracts and produces a droplet that pushes down on the substrate. The rigid substrate pushes back on the droplet, moving its center of mass upwards and causing it to eject. In Ref. 5 we used classical molecular dynamics simulations (MD) to investigate whether it would be possible to control the magnitude of the velocity of ejected Cu liquid droplets by changing the shape and temperature of the initial Cu nanostructure. We found that the more symmetric and hotter the initial nanostructure was, the faster it jumped and moved as a nanodroplet. In 2013, Afkhami and Kondic employed a continuum-model approach to investigate the same issue, and confirmed our results^7^. Recently, Li *et al.*^8^ studied Cu nanostructures deposited on nanopillared substrates and showed that changing the distance between the pillars affected the magnitude of the velocity of the nanodroplets and the time at which they were ejected. Li *et al.*^8^’s work showed a new way of affecting the ejection of metallic nanodroplets, and in this context one wonders whether it would be possible to use those substrates to affect not just the magnitude of the velocity but also the direction at which droplets are ejected. For Leidenfrost droplets^9^ such directional control of ejection is actually possible.

When a droplet is deposited on a very hot surface, a thin vapor cushion develops between the droplet and the surface that protects the droplet from evaporation and makes it levitate. Such a droplet is called a Leidenfrost droplet. By contrast to the classical Leidenfrost regime, droplets in the dynamic Leidenfrost regime experience temporary contacts with the surface and can be affected by the wetting properties of the substrate. In Ref. 9 Agapov *et al.,* threw water droplets onto a hot superhydrohilic substrate made of straight and tilted nanopillared arrays (SNPAs and TNPAs, respectively). For impacts corresponding to the transition boiling regime, where the vapor cushion disappears and partial contact between the water droplet and the substrate occurs (resulting in the formation of a puddle), the droplets were found to rebound with a preferential directionality. The directionality of droplet ejection depended on the type of substrate. When the droplet was thrown onto a SNPA substrate, it rebounded in the direction perpendicular to the substrate. When it was thrown onto a TNPA substrate it rebounded in the direction of inclination of the nanopillars. Preferential directionality was found to be a consequence of the way water wets the substrate: for a SNPA substrate, the puddle spread out symmetrically, and asymmetrically for a TNPA substrate. This asymmetry was carried over during the dewetting process, causing an asynchronous collapse of the puddle that made the resultant droplet to jump in the direction of inclination of the nanopillars.

Inspired by Li *et al.*^8^ work on Cu nanostructures and by Agapov *et al.*^9^ work on Leidenfrost droplets on nanopillared substrates, here we have used MD simulations to investigate whether Cu droplets deposited on nanopillared substrates can be made to jump at desires angles by varying the inclination of the underlying nanopillars.

## Results

**Figure 1:**
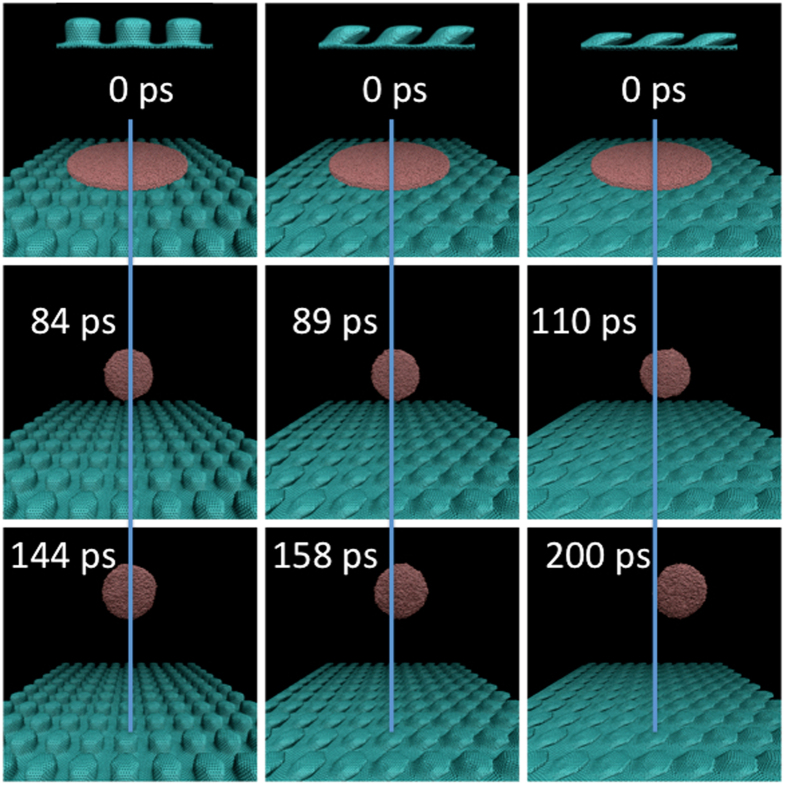
Jumping Cu droplets originated from a Cu disk at 1500 K deposited on three different types of substrates. At the top of each figure, a side view of the substrate is shown. Left column: substrate with vertically oriented pillars; middle and right columns: substrates with pillars tilted 45° and 60^0^, respectively. The blue line is included for reference and marks the vertical *z* axis.

Cu liquid nanostructures in the shape of disks with thickness and diameter of 10 and 120 Å, respectively, and at temperatures of 1500, 1700, 1900 and 2200 K, were created using the same methodology we employed in Refs 5,10. Nanopillared substrates were created by fusing a (30,0) zigzag capped carbon nanotube with a graphene layer using heptagonal rings of carbon. The nanopillars were either vertically oriented or tilted in the *y* direction at an angle *θ* *=* 45^0^, 60^0^. The distance between the pillars, *a*, is 41.24, 41.82 and 42.17 Å for *θ* = 0^0^, 45^0^, 60^0^, respectively; the height, *h*, of the pillars is 22.38, 15.82 and 11.19 Å for *θ* = 0^0^, 45^0^, 60^0^, respectively. The Cu liquid disk and the nanopillared substrate were brought together by depositing the disk at the center of the substrate at a vertical distance of 2 Å. An example of this setup is shown in the upper panels of Fig. 1. Subsequently, a MD simulation was carried out were the disk was let to evolve freely and the substrate was kept frozen. As the MD proceeded, the Cu disk dewets and collapses onto itself, producing a droplet that is ejected from the substrate.

**Figure 2:**
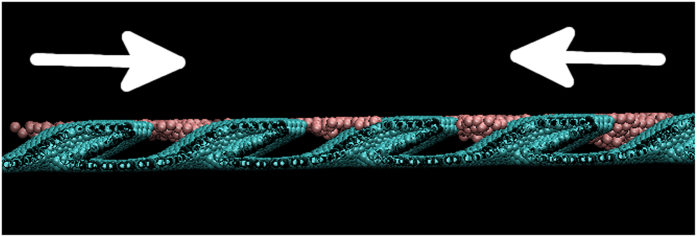
Snapshot of the dewetting process at 10 ps. For clarity, only the Cu atoms above the nanopillars are shown.

The middle and lower panels of Fig. 1 show snapshots taken at different times during the ejection of the Cu droplet. The blue line in this figure is included for reference and denotes the perpendicular direction to the substrate. In the panels of Fig.1, the inclination of the pillars, *θ*, increases from left to right. Clearly, as *θ* increases the droplets deviate farther away from the blue line and in the direction of inclination of the nanopillars. This is explained in Fig. 2, which shows a snapshot of the dewetting process taken at 10 ps at the interface region between the disk and the substrate. For clarity, only the Cu atoms immediately above the substrate are shown. The white arrows indicate the direction of movement of the Cu atoms during dewetting. As it is seen, the Cu atoms moving in the right direction practically slide above the nanopillars. On the other hand, the Cu atoms moving in the left direction are caught in the spaces between the nanopillars (a movie is included in the Additional information). This asymmetry in the movement of the Cu atoms during dewetting causes an asynchronicity in the collapse process, which causes the corresponding droplet to jump to the right, *i.e.* in the direction of inclination of the nanopillars.

**Figure 3:**
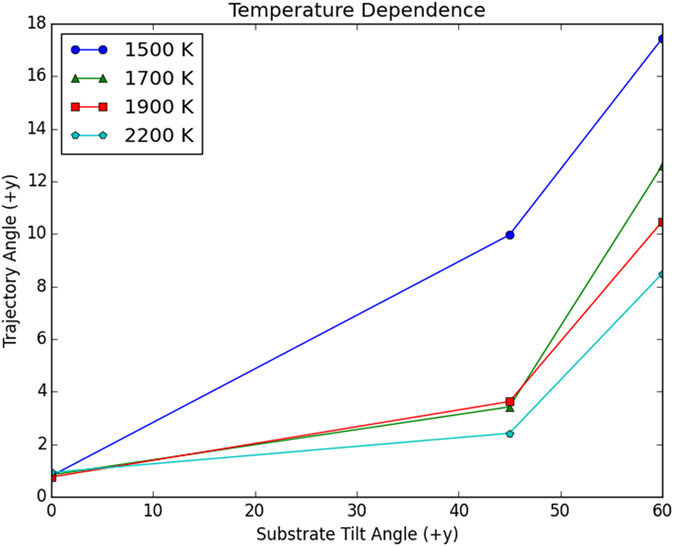
Dependence of the angle of droplet ejection on the substrate’s tilt angle. Four different temperatures are considered. The trajectory angle is the angle that the *y* component of the velocity vector of the droplet makes with the vertical *z* axis. The angles are given in degrees.

We have quantified the direction at which droplets move after ejection by measuring the trajectory angle, which is defined as the angle that the droplet makes with the vertical blue line. As shown in Fig. 3, for the same temperature, the larger is *θ* the larger is the trajectory angle of the droplet. Thus, a Cu liquid disk deposited on a substrate made of C nanopillars can be made to jump in the desired direction by varying the inclination of the nanopillars. For the θ values considered here, *i.e.* 0^0^, 45^0^, 60^0^, the trajectory angle increases with the angle of inclination of the nanopillars; it is expected, however, that when θ = 90^0^, the trajectory ejection angle will become 0^0^.

Interestingly, when the temperature increases the trajectory angle diminishes. For example, in Fig. 3, at 2200 K and *θ* = 45^0^, the trajectory angle is about 2^0^, whereas at 1500 K and *θ* = 45^0^, the trajectory angle is approximately 10^0^. This behavior is explained by the changes in the viscosity of the Cu liquid. When the temperature increases the viscosity diminishes (at T = 1500, 1700, 1900, and 2200 K, the viscosity is 4.288, 3.825, 3.496, 3.149 mPa.s, respectively)^5^, which reduces in turn the asynochronicity of the collapse process. For example, we have found that at 2200 K, the atoms moving left and right both enter the spaces between the pillars (to illustrate this issue, we have included in the Additional information two snapshots taken at 1500 and 2200 K).

**Figure 4:**
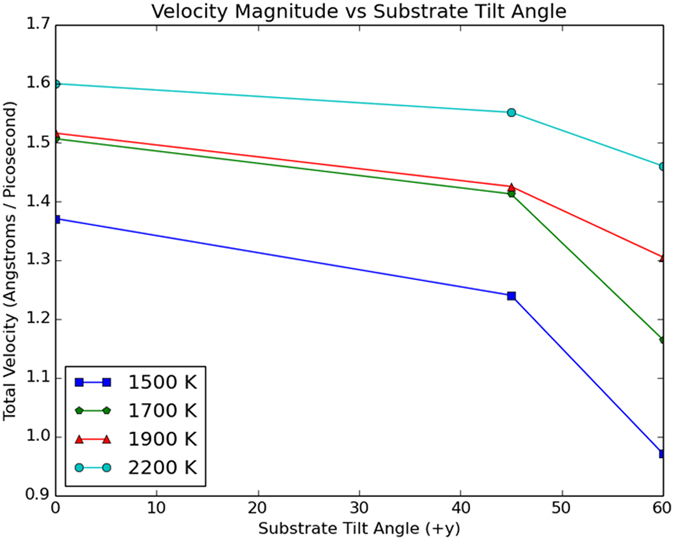
Dependence of the magnitude of the velocity of ejected droplets on the substrate’s tilt angle (in degrees).

It is interesting to delve further into the temperature dependence of droplet ejection by making comparisons between this study and that in Ref. 5 In that work we found that the temperature of a metallic Cu nanostructure that was deposited on a flat substrate affected the modulus of the velocity of the ejected droplet. Does the same dependence occur when the substrate is not flat but nanopillared? Before answering this question, one must be aware of an important difference between the present study and that in Ref. 5 There we considered asymmetric Cu nanostructures, *i.e.* square, equilateral and isosceles triangles. Here we have considered only a symmetric Cu nanostructure, a disk. The disk is placed on a substrate that for *θ* = 45^0^ and 60^0^ is asymmetric. Thus, in the present study it can be said that asymmetry is imposed by the substrate, not by the Cu nanostructure. Despite of this, and as it is seen in Fig. 4, the magnitude of the velocity of ejected nanodroplets does depend on the temperature. Further, to understand whether it is inertia or viscous force the main mechanism driving detachment and ejection of the droplet, we have calculated the Reynolds (Re) number. This is a dimensionless quantity that provides information on the ratio of inertia to viscous forces. For the Cu liquid droplet at 2200 K, considering a characteristic length and velocity of 120 Å and 1.6 Å/ps (*i.e.* the diameter of the Cu liquid circle and the velocity of an ejected droplet, respectively) we obtain a Re ~ 4.5, which compares well with the values in Refs 7,8 This indicates that inertia forces dominate detachment and ejection.

In Fig. 4, for the same temperature, the larger is *θ* the smaller is the velocity; for the same *θ*, the higher is the temperature the larger is the velocity. These findings resemble those we found in Ref. 5 In that work, for the same temperature, the more asymmetric was an initial nanostructure the smaller was the velocity, whereas for a particular initial asymmetric nanostructure, the higher was the temperature the larger was the velocity. It should be noted that Li *et al.*^8^, who considered a Cu liquid on vertically oriented nanopillared substrates, also found a dependence of the ejected velocity with the temperature. In fact their study is similar to the one we present here, except that in their case the nanopillars are not inclined. Nonetheless, they found that as the temperature increased so did the velocity of the ejected droplet. Additionally, Li *et al.*^8^ analyzed how changing the height of the pillars, *h*, and the distance between them, *a*, affected the magnitude of the velocity of ejected nanodroplets. Their *h* varied from 16.67 to 17.22 Å, and it was observed that, within this range, the magnitude of the velocity remain practically constant. In our case *h*’s range of variation is larger, from 11.18 to 22.37 Å, yet for *θ* = 45^0^ and 60^0^ the range considered is very similar to that in Li *et al.*^8^ (*ca* 4 here *vs.* 3.5 Å in Ref. 8). Therefore, for *θ* = 45^0^ and 60^0^ we believe that the changes in the magnitude of the velocity we observe are not caused by the difference in the height of the pillars. As for the effect of *a* on the magnitude of the velocity, Li *et al.*^8^ considered *a*’s that varied between 6.8 and about 13 Å (see Fig. 2b on Ref. 8) and observed that as *a* became larger the changes in the velocity were less and less significant. In fact, it seems that after about *a* = 12 Å the velocity started to reach a plateau^8^. The substrates we considered here are more sparse than those in Ref. 8, with a value of *a* of approximately 42 Å and practically independent of *θ*. In view of this and the results in Ref. 8, we believe that the changes in the velocity observed here are not caused by the differences in *a*. (Quantitative comparisons between the velocities in this study and those in Ref. 8 are difficult to make because the structural parameters of the liquid and the substrates in both studies are different.) Thus, it can be concluded that the magnitude of the velocity of ejected droplets is affected by changes in the symmetry, no matter whether it is the symmetry of the initial Cu nanostructure (as we and Li *et al.* demonstrated in Refs 5,8 for flat and straight nanopillared substrates, respectively), or the symmetry of the underlying substrate (as we demonstrate here) what is being changed. This brings up an interesting question, how does changing both the symmetry of the substrate and the symmetry of the nanostructure affect the velocity of ejected nanodroplets?

To answer this question, we deposited an equilateral and an isosceles triangle, both at 1500 K, on substrates with *θ* equal to 45^0^ and 60^0^. For comparison purposes, we also deposited the same nanostructure on the substrate made of vertical nanopillars (*θ* = 0^0^). For each triangle, four orientations were considered, the original one and three more generated by anticlockwise rotations of 90^0^ around the vertical *z* axis. As before, the nanopillars were inclined in the positive *y* direction.

**Figure 5:**
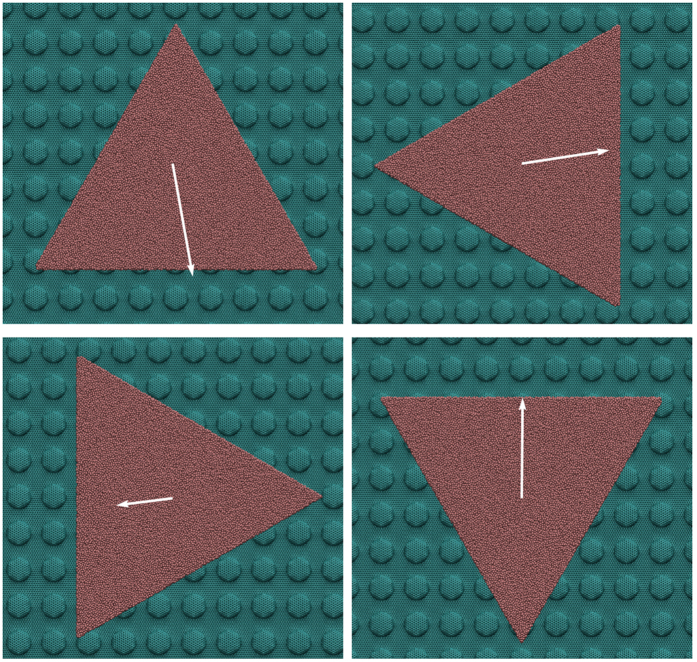
Direction of ejection (as denoted by the white lines) for four different orientations of the equilateral triangle on a substrate made of vertically oriented pillars.

For the equilateral triangle on the substrate with *θ* = 0^0^ we found that the droplets can jump in any direction with no particular preference. This is not what we observed previously for the disk. In that case, for *θ* = 0^0^ the droplet always jumped in the vertical direction. A closer inspection revealed that for the equilateral triangle there is a correlation between its position on the substrate and the direction of movement of the droplet. This can be seen clearly in Fig. 5, where four different initial orientations for the equilateral triangle are considered. The white arrows in Fig. 5 denote the direction and magnitude (size of the arrow) of the velocity of the jumping droplet. The base of the triangle in the upper left panel of Fig. 5 rests on top of a horizontal row of nanopillars. For this orientation, the droplet jumps in the direction which points precisely to where that row is located. (Notice that the nanopillars are inclined precisely in the opposite direction.) In the upper right panel of Fig. 5, the base of the triangle rests on a vertical row of nanopillars, and the corresponding droplet jumps in the direction pointing to where that row is located. Similar observations can be made for the triangles in the remaining two panels of Fig. 5. We do not understand why the triangles jump in this way, yet this result has an important message: in an experiment, for Cu triangles deposited on substrates made of vertical nanopillars, it would be difficult to control in which direction the corresponding droplets will jump, because it seems hardly possible to experimentally precisely control whether the base or the edge of the triangle rests or not on top of a particular row/column of nanopillars. In their study, Li *et al.*^8^ did not discuss whether the equilateral triangles they studied jumped in different directions depending on their relative position to the substrate. It could be that this phenomenon was not observed in their case because the distance between the nanopillars is much smaller than the distance here.

**Figure 6:**
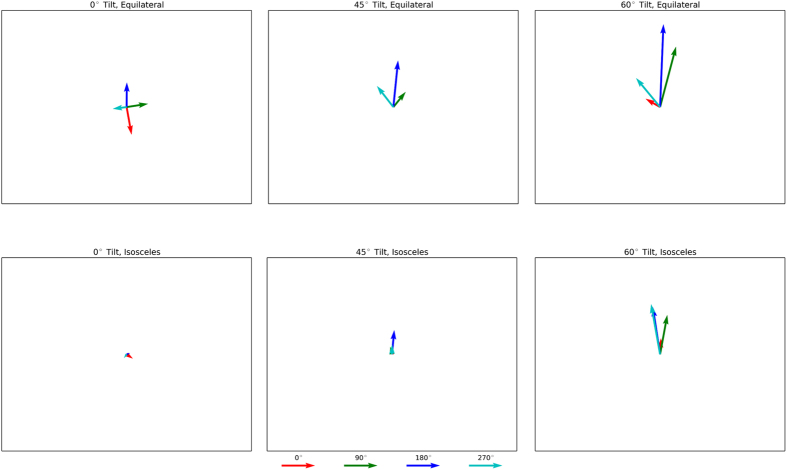
Direction followed by droplets ejected from equilaterals and isosceles triangles deposited on vertical and tilted nanopillared substrates.

The results for the equilateral triangle on the substrates with *θ* = 45^0^, 60^0^ are shown in Fig. 6. Now it seems that it is easier to control the direction of droplet ejection: for this type of substrates, the droplets are found to jump preferentially in the *y* direction. However, there is still a level of uncertainty. For example, for *θ* = 45^0^ and the triangle orientation denoted as 270, there is a significant component of movement in the *x* direction. Yet, after comparing these results to those obtained when *θ* = 0^0^, it can be safely concluded that increasing *θ* washed out the noise effects introduced by the relative position of the equilateral triangle and the substrate, enabling a better control in jumping directionality. (Movies for the ejection of the equilateral triangle on the substrates with *θ* = 60^0^ are shown in the Additional information.) The results for the isosceles triangle are shown in the lower panels of Fig. 6. For *θ* = 0^0^, the isosceles triangle jumps in the vertical direction, as if it were a disk. For *θ* = 45^0^ and 60^0^, the isosceles triangle jumps mostly in the *y* direction with some of component of the velocity in the *x* direction. In fact, preferential directionality is better for the isosceles than for the equilateral triangle. These results are no doubt confusing. Before we had found that for an asymmetric nanostructure, such as an equilateral triangle, there was a degree of uncertainty in the direction in which the corresponding droplet jumped. This seemed to indicate that preferential directionality was easier to achieve if the initial metallic nanostructure was symmetric. However, now we have found that for an even less symmetric nanostructure, *i.e.* an isosceles triangle, preferential directionally is easier and not more difficult to achieve, as one would have expected from the results on the equilateral triangle. In view of all of these results one can only safely conclude that for the purpose of experimentally controlling the direction of droplet ejection, it is advisable to change the symmetry of the substrate and not the symmetry of the deposited metallic nanostructure. That is, it is advisable to consider symmetric nanostructures deposited on asymmetric substrates.

Afkhami and Kondic^7^ used a continuum-model approach to investigate the dewetting of Au triangles on flat substrates and found that upon collapse, the triangles produced droplets that were ejected not perpendicularly to the substrate but at an angle. This angle of ejection, equivalent to what we call here the trajectory angle, changed with the contact angle. Experimentally, the contact angle of a liquid on a substrate is measured through the liquid where the liquid/vapor interface meets the solid substrate. The contact angle quantifies the wettability of a solid by a liquid. The less wetting is the substrate, the larger is the contact angle. Afkhami and Kondic^7^ varied the contact angle by varying the wettability, and found that the more wetting was the substrate (*i.e.* the smaller was the contact angle) the larger was the trajectory angle of the ejected droplet. Changing the metallic liquid, the substrate or both can vary wettability. For example, one can use Cu instead of Au, or graphite instead of Si. Here we show that it is not necessary to change the materials that make up the droplet and the substrate, but instead one can change the structure of the substrate. That is, preferential directionality of droplet ejection can be achieved by using nanopillared substrates with different inclinations, in a similar fashion to what Agapov *et al.*^9^ did for Leidenfrost droplets.

It is a good moment now to compare our results to those of Agapov *et al.*^9^ They throw water micro-droplets on a hot superhydrophilic nanopillared substrate. When the droplets contacted the substrate, they flatten out and formed a puddle. The disappearance of the vapor cushion due to the impact and the subsequent contact between water and the superhydrophilic substrate caused the flattening of the droplet into a puddle. Spreading of the puddle depended on the inclination of the nanopillars. For vertical nanopillars, spreading of the puddle was symmetric whereas for tilted nanopillars it was asymmetric. These puddles subsequently underwent dewetting, collapsing into droplets that were ejected with a trajectory angle that was negligible for symmetric puddles and significant for asymmetric ones. In Agapov *et al.*^9^’s experiment, during detwetting the droplet becomes a Leidenfrost droplet, which means it levitated above the substrate. Thus, in this experiment the asynchronicity of the collapse process could not be caused by the way the atoms move relative to the underlying pillars upon dewetting, *i.e.* by whether the atoms slide above the pillars in one direction and were caught between the pillars in the opposite direction. In Agapov *et al.*^9^’s experiment asynchronicity of the collapse process is caused by the asymmetry of the initial puddle. In our case, asynchronicity is caused by both the asymmetry of the initial Cu puddle and by the way that upon dewetting the Cu atoms move relative to the underlying nanopillars.

**Figure 7:**
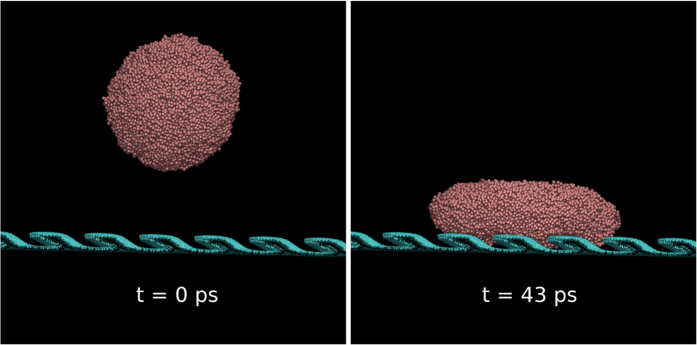
Left: a Cu droplet thrown at a 60^0^ tilted pillared substrate at 300 m/s. Right: Cu droplet flattens out once it has impacted the substrate, but it does not form an asymmetric puddle.

For completeness, and inspired by Agapov *et al.*^9^ study, we decided to investigate whether Cu droplets can be made to rebound at an angle if they were to be thrown towards a nanopillared substrate. To our knowledge this issue has not been investigated yet, although there is a related work by Boneberg *et al.*^11^. They showed that nanosecond laser pulses could be used to eject nanodroplets from a flat substrate, which then were collected in another flat substrate place immediately above. The nanodroplets originated from an array of nanostructures that had a different degree of order. This degree affected the size distribution of the collected nanodroplets. For ordered arrays, the nanodroplets collected had a monodisperse size distribution, whereas for less ordered arrays big and small nanodroplets were collected. Big nanodroplets were found to spread out upon impacting the collecting substrate. Boneberg *et al.*^11^ did not discuss the symmetry of the spread out structures. They did not discuss either whether the droplets rebounded after being collected. We have used MD simulations to throw Cu droplets onto the vertical and the 60^0^ tilted graphene nanopillared substrates, see Fig. 7. Cu droplets at 1500 and 2200 K were thrown at speeds of 100, 200 and 300 m/s. Upon impact, their wetting behavior was investigated visually and quantitatively by comparing the profile of the spread out droplet to that of a perfect circle. We found that in all the cases studied, the droplets did not spread out significantly, and therefore we could not see a significant difference between the profile of the spread out droplet and that of a perfect circle. Rebounding only happened at impacting velocities of 200 and 300 m/s, but the rebound was always perpendicular to the substrate. We found that to make the droplet rebound at an angle, the velocities must be ridiculously large (*i.e.* 1 km/s, which produces a trajectory angle of 11^0^). These results indicate that the Cu-C system is not the appropriate for extending Agapov *et al.*^9^ experiment to metallic nanodroplets. One must instead chose a metal with a smaller surface tension and a substrate that interact more strongly with it.

## Discussion

We have used classical molecular dynamics simulations to show that liquid Cu nanostructures can be ejected at desired angles by depositing them on substrates made of tilted C nanopillars. The direction of ejection coincides with the inclination of the nanopillars, and this is a consequence of the asynchronicity of the collapse process that happens upon dewetting. Control however is not always possible: the relative position of an asymmetric Cu nanostructure and the underlying nanopillars can affect significantly the ejection angle. This suggests that if laser-induced dewetting of metallic nanostructures on nanopillared substrates were to be used to control the direction of ejected nanodroplets, it would be advisable to consider metallic nanostructures that are symmetric. We also suggest that the ongoing research on Leidenfrost represents a rich area from which to borrow ideas for controlling the movement of metallic nanodroplets, and that perhaps many of the experiments on Leidenfrost droplets could be extrapolated to metallic nanodroplets. However, care must be exercised in doing such extrapolation. Indeed, we have found that the Cu-C system is not suitable for extending Agapov’s *et al.*^9^ experiment on Leidenfrost droplets in the dynamics regime to metallic nanodroplets, and that instead a droplet that has a smaller surface tension and or a system that interacts more strongly should be considered.

## Methods

Cu nanostructures in the shape of a circle, triangle and isosceles triangles were extracted from Cu bulk liquid samples that were created at 1500, 1700, 1900 and 2200 K using the Embedded Atom Method (EAM) potential. The extracted structures were then deposited on graphene nanopillars. The graphene nanopillar structures were described with the Adaptive Intermolecular Reactive Empirical Bond Order (AIREBO) potential^12^. The interaction between the Cu liquid nanostructures and the graphene nanopillars was described with a Lennard-Jones potential that was fitted to reproduce experimental and theoretical data as described in Ref. 10. Once deposited, the Cu liquid nanostructures were led to evolve freely whereas the substrates were kept frozen. All the calculations were performed with the software LAMMPS^13^.

## Additional Information

**How to cite this article**: Horne, J. E. *et al.* Extrapolating Dynamic Leidenfrost Principles to Metallic Nanodroplets on Asymmetrically Textured Surfaces. *Sci. Rep.* **5**, 11769; doi: 10.1038/srep11769 (2015).

## Supplementary Material

Supplementary Video 1

Supplementary Video 2

Supplementary Video 3

Supplementary Video 4

Supplementary Video 5

Supplementary Information
