# Supplementary material for: Extrapolating Dynamic Leidenfrost Principles to Metallic Nanodroplets on Asymmetrically Textured Surfaces
Source: Sci Rep. 2015 Jun 30;5:11769. doi: 10.1038/srep11769 (PMC4485316; doi:10.1038/srep11769)
Supplement: Supplementary Information [file srep11769-s6.pdf]

Additional Information

Extrapolating Dynamic Leidenfrost Principles to Metallic Nanodroplets on Asymmetrically Textured Surfaces

*Joseph E. Horne[1], Nickolay V. Lavrik[2], Humberto Terrones[1], and Miguel Fuentes-Cabrera[2,3]**

1 Department of Physics, Applied Physics & Astronomy, Rensselaer Polytechnic Institute, 110 Eighth Street, Troy, 12180 NY, USA

[2]Center for Nanophase Materials Sciences, Oak Ridge National Laboratory, Oak Ridge, TN, 37831, USA

[3]Computer Science and Mathematics Division, Oak Ridge National Laboratory, Oak Ridge, TN, 37831, USA

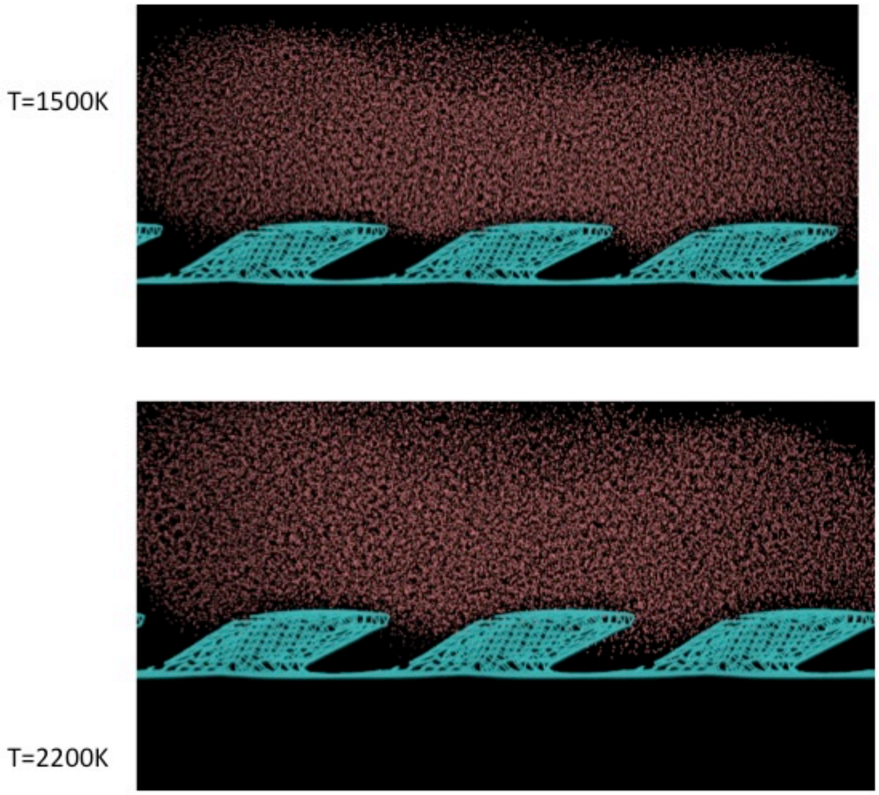

Figure captions

FigS1. Two snapshots showing the dewetting process at T=1500K and T=2200K. At T=2200K, the Cu-atoms enter the spaces between the pillars more than they do at T=1500K, and do it so whether moving in from the left or the right.

Video Legends

Complementary-movie-for-Figure2.avi Movie showing the movement of atoms as the Cu-liquid nanostructure dewets. The movie shows the interface region close to the substrate.

Equilateral-triangle-60substrate-0rotation.avi. Equilateral triangle rotated 0 degrees on a nanopillared substrate made of 60 degrees inclined pillars.

Equilateral-triangle-60substrate-90rotation.avi. Equilateral triangle rotated 90 degrees on a nanopillared substrate made of 60 degrees inclined pillars.

Equilateral-triangle-60substrate-180rotation.avi. Equilateral triangle rotated 180 degrees on a nanopillared substrate made of 60 degrees inclined pillars.

Equilateral-triangle-60substrate-270rotation.avi. Equilateral triangle rotated 270 degrees on a nanopillared substrate made of 60 degrees inclined pillars.
